# *“She held my hand and advised me”*: Young migrants’ experiences of individual peer support to access health and social services in two small towns in southwestern Uganda

**DOI:** 10.1371/journal.pgph.0003713

**Published:** 2024-11-22

**Authors:** Edward Tumwesige, Rachel Kawuma, Allen Asiimwe, Patricia Nabimanya, Stella Nakate, Sarah Bernays, Janet Seeley

**Affiliations:** 1 Social Sciences, MRC/UVRI & LSHTM Uganda Research Unit, Entebbe, Uganda; 2 School of Public Health, University of Sydney, Sydney, Australia; 3 Department of Global Health and Development, London School of Hygiene and Tropical Medicine, London, United Kingdom; 4 Social Science Core, Africa Health Research Institute, KwaZulu-Natal, South Africa; TAPMI at Bengaluru: T A Pai Management Institute Bengaluru, INDIA

## Abstract

We describe how a pilot intervention called "Lending a Hand" was implemented to mitigate some of the risks associated with migration among young recent migrants (14–24 years) in two small towns in south-western Uganda. The design of the intervention was informed by the `protection-risk framework’, with key protection components of the intervention (positive role models/ “good” social network, safer environment, health and social support) affording support to young migrants to counter risks in their new environment. As part of the intervention (November 2021-January 2023), peer supporters were recruited and trained to provide practical assistance, emotional support, and guidance to young recent migrants. We conducted qualitative in-depth interviews with 20 young migrants (11 males and 9 females). They were purposively selected to participate in two in-depth interviews each to explore their experiences with peer support. Young migrants were eligible to participate if they were aged between 14 and 24 years and in their first year as a migrant in the town. Data were analysed thematically, and three themes on the role of peer supporters were identified based on the protection-components drawn from the protection-risk framework: facilitating access to health services, offering responsive and person-centred support and fostering a social support system, friendship and mentorship. We found that peer supporters improved young migrants’ access to health and social support. They facilitated access to healthcare services, provided information and counselling services and offered responsive and person-centred support. Peer supporters in the Lending a Hand intervention played a valuable role in addressing healthcare challenges faced by young migrants. This experience offers lessons for the integration of formal peer support into interventions targeting young migrants to access health and social support services.

## Introduction

Young people are increasingly leaving their family homes in search of work opportunities [1, 2], education [3], security [4], and improved living conditions [5] as they transition into adulthood. In sub-Saharan Africa, cross-border migration is common, with young people comprising a significant proportion of international migrants [6, 7]. In Uganda, as in many other countries in Africa, in addition to international migration, internal rural-urban migration is prevalent [8–10]. Young migrants, even those moving within their own country face challenges as they move, including exposure to infections [11], psycho-social issues [12], precarious living conditions [13, 14], and exploitation and abuse [9, 15]. Young migrants may struggle to access healthcare [16, 17], education [18], and social support systems [14].

The “Lending a Hand” intervention, was co-designed with participants who took part in a formative study with young migrants in South Africa and Uganda [19, 20]. Our findings from that study revealed that very few young recent migrants felt able to access formal health services to support them to manage their sexual and reproductive health risks. The study participants suggested that having someone to support new migrants in navigating the services in a new setting would help them to access the healthcare services, which led to the co-design of an intervention based on the provision of peer supporters and a telephone helpline.

The Lending a Hand intervention was implemented from November 2021 to January 2023 and aimed to test a protective support structure for young migrants (aged 14–24 years old) in two small towns in south-western Uganda, as a means of early intervention to reduce the harm of patterns of risk behaviour (such as condomless sex, over-use of alcohol) associated with adolescent and young people’s migration. The aim of this was to assess whether the intervention was possible to be undertaken (feasible), would be embraced by young migrants (acceptable) to mitigate the risks that may be associated with migration.

The young migrants were connected to the intervention through the peer supporters (three males and two females), who had themselves previously been migrants, but who were now settled in the communities.

In this paper, we examine how young migrants in Uganda benefited from peer support to access health and social services provided by the Lending a Hand intervention.

### Theoretical framework

We developed a theory of change to guide us in evaluating the intervention, based on the protection-risk framework by Kabiru et al. [21]. The protection-risk framework was drawn from the ‘Problem Behaviour Theory’ by Jessor et al. [22] and its underpinnings describe that in society, there are risk factors that are likely to cause people to develop problem behaviour. For instance, in the case of young people, it could be problems such as drinking excessive quantities of alcohol, use of recreational drugs and engaging in sexual behaviour which poses a risk of acquiring HIV or other sexually transmitted infections. Following the protection-risk framework protective factors can be put in place to address the risks. Protection includes familial and peer role-models who might promote prosocial behaviour; controls protection includes individual-level factors such as religious faith or the presence of family/older friend/trusted adult. Support protection includes contextual support like peer networks, fellow-workers that promote pro-social/health enhancing behaviour. Thus, enhancing the `protection’ factors through harm reduction interventions should moderate the impact of the risks in a particular context by providing positive role models/ “good” social network, a safer environment, and access to health and social support. The Lending a Hand intervention framework has been described elsewhere [23].

## Methods

### Study design and setting

We conducted a descriptive qualitative study with some young people (aged 14–24 years), who were part of the Lending a Hand intervention in two small towns in Kalungu district, southwestern Uganda. The first town is located along the highway that connects Uganda to the Democratic Republic of Congo, Rwanda, and Tanzania while the second is a small trading centre, located about 20 kilometres away from the first. The main ethnic group in the study areas are Baganda but there are other tribes such as Banyankore, Bafumbira, and Banyarwanda. There are low-cost housing units (often single rooms) available for rent in both towns. Young people make a living in these places through vending goods on the street, providing labour in markets, building sites and farms, working in motor vehicle garages, restaurants and bars, and undertaking fishing-related activities.

### Implementation of ‘Lending a hand intervention’

Before implementation of the Lending a Hand intervention, we undertook a baseline assessment of young migrants’ needs and designing of the intervention with participants (young migrants) and service providers/local leaders from January 2020 to April 2021. This included conducting engagement meetings with stakeholders in the communities to tell them about the study and gather their views on the intervention design. To assess the needs of the young migrants, we conducted a cross-sectional survey between November 2020 and March 2021. This survey targeted a group of young individuals aged 14 to 24 who had migrated to each town within the past nine months, a period which we chose based on our formative work as being sufficient for young migrants to learn and adapt to experiences in their new environments [20]. The survey aimed to gather information about their duration of stay in these areas, their current occupations, sexual and reproductive health needs, health seeking behaviours as well as their mobility patterns and experiences. While conducting the survey, all the young people who participated were given information about the planned intervention and those who expressed interest in joining the intervention were encouraged to do so.

After the baseline, we recruited staff in preparation for the intervention. These included a study counsellor, one administrative assistant and five peer supporters. In addition, a memorandum of understanding was signed between the study and one government health facility in each town to support the young migrants who may be referred. At each of the facilities, two health workers trained in adolescent related care services were identified as focal persons with whom the study team worked closely by referring young people who needed health related support during the period of the intervention. The health workers were paid a monthly stipend equivalent to ~68 USD each to support this work. In addition, the study counsellor visited each facility on rotational basis and offered support to any young migrant who needed services. A government health facility was preferred because government usually equips these facilities with drugs which people can access at no cost and there are additional sexual and reproductive services also provided at no cost. Besides the staff, a toll-free telephone, managed by the study administrative assistant was installed and publicised in the communities for young migrants to call if they needed any support. Those with health-related needs could talk to the study counsellor on the telephone who either supported them or referred them to the health facilities for further care. The inclusion of the toll-free telephone line in the study design was a modification to provide ‘virtual’ support because of COVID-19 disruptions that saw lockdowns in Uganda (March-September 2020) which restricted face to face interactions and meant that an original plan to provide a drop-in centre in each town could not be executed.

To create awareness about the intervention in both communities, a van with a loud-speak system was deployed in the two settings, broadcasting information detailing wat the intervention was about. In additional, posters with details about the intervention including contact information were displayed at various places frequented by young people such as bars, sports betting facilities and markets.

### The peer supporters

Five young people (aged between 18–30 years) who had previously migrated to these towns and had settled in the community, which gave them first-hand experience of migration and the challenges, solutions and opportunities were recruited, as peer supporters for the intervention. They included three males and two females, who had previously been doing manual labour such as fishing boat crew, casual farm labourer, commercial motorcycle rider, and for the females sex work as part time job. Before taking up the role of a peer supporter they underwent a comprehensive three-day training programme conducted by the project staff in Luganda, a local language commonly used in the two settings. During the training they were given detailed information about the study, were trained in lay counselling, community mobilisation and engagement, and referral mechanisms as well as learning some basic research methods. The study counsellor briefed them about the services offered at the health facilities free of charge to enable them to provide accurate information to young migrants about available services and the referral system process. After the training, each peer supporter was provided with pens and books to document details of the young migrants they met. Peer supporters were provided with a phone and monthly airtime to regularly communicate with the research team (co-authors ET, AA, RK, PN and SN) and young people. They also met young people face to face where possible.

All the peer supporters were employed as project part-time staff and paid a monthly salary of 400,000/ = Uganda shillings (equivalent to ~108 USD) to dedicate time to the project. This amount covered their transport costs and incidental expenses as they engaged in the project related activities. It was not sufficient to cover their other expenses such as rent, food, fees for their children, and utilities like water and power in town. Given this, peer supporters continued with their other work but dedicated some time to the project when needed. To maintain regular contact with the research team (as above) and the peer supporters, besides periodic physical meetings, a WhatsApp group was created through which frequent communication was carried and solutions suggested to any challenges in real time by members of the group.

### Sampling and recruitment for the qualitative study

Over the course of the intervention, lasting 15 months, the peer supporters assisted 385 (176 males and 209 females) young migrants to join in three main ways: they encouraged those who wanted to join to call and speak to the study team using the toll free telephone line, or directed them to the health facilities to talk to the intervention focal health workers and, for some they physically escorted those who could not go alone to the health facility either because they did not know there or could not speak the local language and therefore needed an interpreter. Between March and April 2022, we purposively sampled out 20 (11 males and 9 females) from the 385 young migrants in the intervention to take part in a qualitative study.

The purposive criteria used included selecting young migrants who were already enrolled in the intervention. We also considered other sampling characteristics such as gender, age, location, and the nature of work they did so we were able to understand the intervention’s impact across different young people in each setting, as these were people who were able to provide appropriate and useful information on the migration experience and their knowledge of the intervention [24].

Recruitment for the qualitative interviews as in the intervention was done by the members of the research team (the two social scientists, ET and AA, who carried out all the data collection) and the peer supporters. The research team members shared a list of the young people sampled for the interviews and these were approached by the peer supporters either physically at their workplaces, homes and places they frequented, or through a phone call to invite them for an interview.

### Data collection

We aimed to conduct two in-depth interviews with each of the twenty young people. The first interview, held within the first month of their enrolment, sought to explore their experience of daily life, including mobility, income generation, and access to health services. The second interview, after a period of six months of being enrolled in the intervention, captured any changes that could have happened since the first interview. While all the 20 young people were interviewed for the first interview, for the second interview, only 14 (10 males and four females) were available for the interview because the rest had moved away from town, and they could not be contacted either physically or by phone.

The interviews were conducted by two social scientists (ET and AA) with experience in qualitative data collection methods. A list of topics, covering migration history and experience of settling into the new place was used to guide the conversation. All the interviews were conducted face-to-face at the participant’s choice of place and were mainly conducted in the local language (Luganda). Interviews were audio recorded with participant consent. A few interviews were conducted in Rufumbira, a language used by migrants from Rwanda and Kisoro district in the far south-west of Uganda, by one of the two social scientists (ET) who spoke both languages. Each interview lasted up to one hour. The interviews were transcribed verbatim into a word document and translated to English. The transcripts were reviewed by the lead social scientist throughout the course of the study to check for consistency and to ensure that meaning was not lost during the transcription. Final interview transcripts were kept on a secure server. All audio-recordings were deleted after transcription.

### Data analysis

A manual thematic content analysis approach was used to distil key concepts from the interviews. We followed the six steps for thematic analysis set out by Braun and Clarke [25]. Two team members (ET and AA) conducted the first level of manual analysis by carefully reading through two scripts and identified common codes. The identified codes were then shared and discussed with the rest of the study team (JS, RK and SB), and these were used to develop a coding framework (S1 Table). Codes with similar or close meanings were grouped together to generate broader themes. Data were charted on an Excel matrix under the relevant themes to provide an overview and enable quick and easy navigation. All the charted data were also reviewed by the lead social scientist (RK). After discussion among the team about the findings, the two interviewers (ET and AA) led on preparing analytical memos distilling the results on the key themes on which this paper draws the experiences of young people as a protective mechanism for young migrants navigating new urban environments. The decision to focus on peer support was informed by its relevance to the Protection-Risk framework which shaped the Lending a Hand intervention. Peer support was an integral part of the intervention.

### Ethical considerations

Ethical and research approval were granted by the Research Ethics Committee of the Uganda Virus Research Institute (GC/127/20/06/746) and the Uganda National for Science and Technology (SS 5116). Participants were approached and invited to take part by the local research team and asked to provide written consent to participate in the study activities. We followed Uganda National Council for Research and Technology guidance [26] which provides for participants aged 16–17 years old, living independently and providing for themselves financially, to be considered emancipated minors and able to give their own consent without requiring approval from a parent or guardian.

## Results

### Participants

As described above, 20 young people (11 males and nine females) took part in the interviews. In terms of education, 12 had reached secondary level, with two attaining a tertiary level certificate following further training, one in electrical installation and another in tourism and travel. Three of the young women worked in bars and restaurants, while the young men usually held more than one job in construction, roadside vending, and loading vehicles in markets or undertaking metal work. Others were engaged in fishing and serving in shops. Additionally, three young people, including two students and one female who described herself as a housewife were categorized as having no paid employment. Among the participants, four lived with other family members. Two participants, a male and female, lived with their partners and children: one with a single child and the other with two children. One male participant lived with his two children from a previous marriage and another male participant lived with his uncle. Five participants lived alone, and the remaining 11 participants lived with either a friend or colleagues at workplace (Table 1).

**Table 1:** Characteristics of study participants in the ‘lending a hand’ qualitative study conducted between March-November 2022 in southwestern Uganda.

ID no	Gender	Age	Education level	Relationship status	Months spent in town at 1st interview	Work
P01	Female	22	None	Single	4	Sex work, Bar, and restaurant attendant
P02	Male	20	Secondary	Single	8	Multiple jobs (Construction labourer and farm labourer)
P03	Male	16	Primary	Single	8	Unemployed (Student)
P04	Male	20	Primary	Single	5	Fishing
P05	Male	18	Secondary	Married	4	Multiple jobs (Farm labourer & loading goods in the market)
P06	Female	22	Primary	Married	2	Unemployed (`housewife’)
P07	Male	22	Primary	Single	3	Rice Factory labourer
P08	Male	23	Secondary	Single	4	Unemployed
P09	Female	22	Secondary	Single	4	Unemployed (student)
P10	Female	18	Secondary	Single	4	Shop attendant
P11	Female	17	Secondary	Single	1	Restaurant Waitress
P12	Female	20	Secondary	Single	9	Multiple jobs (Laundry, and farm labour)
P13	Female	20	Primary	Single	2	Waitress in a restaurant
P14	Male	19	Secondary	Single	8	Multiple jobs (laundry, house cleaning, waitress)
P15	Female	20	Secondary	Single	9	Multiple work including sex work
P16	Male	18	Primary	Single	6	Multiple jobs (Vending soft drinks, offloading goads, and construction labourer)
P17	Female	17	Secondary	Single	7	Bar and restaurant attendant
P18	Male	21	Secondary	Married	3	Rice Factory labourer
P19	Male	21	Primary	Single	8	Rice Factory labourer
P20	male	21	Secondary	Married	10	Selling sim cards

We organise our findings below by the three themes related to the role of peer supporters namely: facilitating access to existing care, offering responsive and person centered support and fostering a social support system, friendship and mentorship. The quotations within the text are taken from the translated transcripts from the interviews.

### Role of peer supporters in facilitating access to existing care

This theme explored the key role of peer supporters in enhancing young people’s access to health care services and the sub-themes provide a detailed understanding of how peer supporters contributed to improving healthcare access and addressing challenges faced by young migrants.

*Facilitated access to health care services*. Young people acknowledged the pivotal role played by peer supporters in helping them access and utilize healthcare services. Young people who had previously experienced lengthy waiting times at the health facilities noted improvements after engaging with the intervention and the peer supporters. One young, 19-year-old, expressed frustration with his prior experience when trying to access care from the local clinic:

*There is a way those health workers used to behave. Before I joined [your intervention] I never thought of going back* [*to the health facility] again. You can go there as early as 7 a.m. and you will sit until 1 p.m. before you are seen.*

During the intervention, peer supporters contacted health workers in advance or as participants moved to the health facility, facilitating their navigation through the system, something young people appreciated to significantly reduce on the waiting time. A 22-year-old young woman commented on the impact of peer support on waiting time:

*They [health workers] treated me well I will not lie. They attended to me so fast. When I reach there, I don’t sit there for so long. What has made me happy is that when you arrive there you simply pass and meet a health worker*.

Another young person reflected on the contrast between her prior and current experiences and the positive effects of being supported through the intervention:

*When I had just arrived in [in town], barely a month, I got seriously sick and could hardly manage to stand on my feet. When I went to the health facility and tried to explain to a health worker how much pain I was feeling she just shouted at me to sit and wait. She asked if the other people who were seated were not patients, but the fact is I was very badly off. But today before you even reach the facility there is already someone there knowing that you are coming and is going to support you and they speak so well. They ask you politely and do not shout at you as if you quarrelled with them. That is what they call customer care.* 22-year-old female.

In addition, peer supporters accompanied young people who felt apprehensive about navigating healthcare procedures alone or directed them to the health workers who collaborated with the lending a hand intervention and had been taught about the specific challenges young people face in accessing healthcare, which promoted a more supportive environment. A 17-year-old girl, shared her experience when she fell ill, stating:

*I did not have money to go to the health centre when I got sick and I remembered [peer supporter], I said let me go and see her, and good enough when I told her she responded by escorting me to the health centre and I was treated for free*.

Some participants faced challenges with stigma when accessing certain services, such as obtaining condoms, which peer supporters mitigated by offering discreet support, giving young people condoms in a private setting. A 21-year-old male remarked:

*She [a peer supporter] helped me to access a box of condoms. You know we are always scared of people seeing us carrying condoms but she put them in a small black polythene bag and gave them to me*.

When describing his experience, a participant living with HIV recounted how crucial peer support was to him accessing HIV care. When he arrived in the town, he wanted to find a health facility where he could enrol for HIV care. A few friends he had made referred him to the peer supporter who helped him obtain the necessary transfer documentation:

*…. my friends took me to the community youth leader [Peer supporter] who helped me to get treatment at health facility xxxx in town one. He helped me to get the local council introduction letter that introduced me to the health facility as a resident [of the area] and when I presented it and the documents from my previous health facility I was enrolled.* 20-year-old male

Young people highlighted a range of critical services they received through peer support. A 22-year-old young woman who worked as a sex worker and restaurant attendant recounted one example of the support she had received:

*I got the disease I told you about (pains in the private parts and discharge). When I got that disease, I told my friend and she brought me to her [peer supporter] and she explained to me what the intervention is about and after we went [to the health facility] where she helped me to get a tube [of treatment] free of charge*.

Peer supporters acted as interpreters and translators for young people facing language barriers, which improved communication for them with the health workers. An 18-year-old male, working in a rice farm said:

*…I would speak a different language and he would translate for them [health workers] in the local language and then he would also translate back for me whatever they said. He did almost the same when we went there to the healthy facility [for circumcision].*

During the intervention peer supporters notified health workers in advance about young people who were coming to the health facility ensuring that they were promptly attended to. This allowed them to quickly return to their work activities and encouraged other young people to seek health services. However, some young people felt uncomfortable with this ‘additional support’ in accessing health services as it meant meeting a health worker ahead of those who had arrived earlier. A 21-year-old male expressed his perspective on this:

*I remember she [peer supporter] told me that whenever I go there I should always introduce myself to the health workers so they can attend to me in time but that is not me, I don’t want to be seen before the people I found there who came so early. So, I gave it time and I was attended to after the people I found there had been attended to.*

*Provided information and counselling services*. In addition to facilitating access to health care services, peer supporters played a crucial role in offering relevant information on health and counselling tailored to young migrants’ needs. A 22-year-old participant and mother of two, who had newly been introduced to pre-exposure prophylaxis (PrEP) for HIV prevention at second interview, attributed her knowledge and access to a peer supporter. The peer supporter, who had previously worked as a liaison person between organizations that supplied PrEP to sex workers had personal experience using it for HIV prevention and recommended PrEP to the young woman due to her elevated risk of HIV acquisition as a sex worker. The participant stated:

*I talked to her [the peer supporter]. She also told me a lot of information about PrEP and how it is used and she helped me to get it. She escorted me to xxxx health facility where I got it from.*

Peer supporters, equipped with basic counselling training, provided emotional and guidance on navigating various life challenges, such as helping young people in managing workplace conflicts as well as managing finances. One young woman explained that she was one of the beneficiaries:

***…****if I have done something wrong or my workmates have lied against me to our boss (employer) I leave the workplace feeling so bad. I share with her [peer supporter] then she counsels me, she tells me how to deal with them, keep silent, and do my work well as expected. From there I leave her place when I am settled and at peace with them.* 17-year-old female.

For some young people, they shared with their peers some of the guidance and advice received from the peer supporters, especially on strategies for overcoming challenges.

*I tell them [friends] that they have to be careful in this place because things are not easy, they have to be careful with their lives and learn to keep and make use of the money they earn however little it is. I also remind them that they have to be firm with the situation of which everything seems to be very expensive and they have to balance their money as they spend on food. About their life, they have to enjoy it but not to the maximum because you may have a job today and later on, you are laid off.* [Male, 21-year-old]

A 19-year-old young woman emphasised the impact of peer support in helping her to help others in the future:

*I have learnt to be helpful or supportive because it clearly shows that even those who started the intervention have good hearts and intentions of helping when they don’t know where the kind of people they are supporting come or came from. I have therefore learned to care about others irrespective of who and where they come from.*

Young migrants who engaged with the peer supporters found that their access to health services was more straightforward than it had been before the intervention.

### Role of peer supporters in offering responsive and person centred support

Young people expressed their satisfaction with the flexibility of the peer supporters’ assistance, accommodating their unique situations. They mentioned how they were not always able to access services or keep appointment times because of work commitments, yet the peer supporters remained consistently available and easy to contact. One 21-year-old male described how he regularly visited the peer supporter’s home to get condoms for himself and his friends.

*…this kind of arrangement has helped bring services near to us because sometimes you need condoms and going to health centre to access them is not easy but when you come to the peer supporter you get them so fast. When she does not have them she asks you to wait until when she gets them the next day and she brings them to your place. The peer supporter is easy for us to approach and she is also fast to give us what we need.*

Participants who had not yet benefitted from the peer’s support expressed optimism that they would also receive support when needed. One young woman, aged 19, stated: *…I have not fallen sick now*. *With this*, *if you fall sick*, *you just go and the peer supporter will link you to the health facility for treatment*.

Another young man aged 22 years old said:

*I now know that if I get sick for example from malaria, I will tell my cousin brother to call the peer supporter to bring me here. I wrote the peer supporter’s number on a piece of paper so I can use it whenever I get sick.*

The accounts of prompt assistance highlight the practical benefits of peer support in addressing time-sensitive situations.

### Fostered a social support system, friendship and mentorship

Peer supporters fostered friendships with young migrants and provided a supportive network akin to familial relationships. Participants valued the understanding and guidance offered by peer supporters. A 20-year-old male shared:

*I went to tell her that I was happy about her guidance and support and since then we have been friends. She advises me on many things especially keeping myself safe.*

In addition to residing in the same community as the peer supporters, some young people revealed that they found it helpful to connect with individuals who were once migrants like themselves. They considered peer supporters to have the qualities of *‘friendliness and helpfulness’* as illustrated in the following quote from a 21-year-old man: *You may fear going to the health facility but here she is our friend and she understands our situation and she makes everything easy for us*.

Some young people noted how they felt at ease sharing their emotional and stressful experiences and finding solace in the support provided by the peer supporters. Life in the new place, they explained, presented numerous challenges that often caused worry. Sharing with peer supporters who had traversed similar paths offered them solace and renewed optimism for the future.

*They [peer supporters] have been so supportive in terms of restoring young people’s hopes for those that have been hopeless. They have also linked us to people who have encouraged and advised us in one way or another.* [19-year-old male]

A 20-year-old migrant who engaged in commercial sex work recounted her experience with peer support and the relief she got after sharing with a peer supporter what she was going through in her sex work business.

*I felt so relieved after that discussion with her because I was full of fear about how I would move on with life while having such difficulties with young men who refused to wear condoms yet I wanted money so that I would be able to get what to eat.*

Some young people even likened peer supporters to parental figures in their lives who listen and genuinely care for them. A 20-year-old woman commented:

*Let me say that they wish so much for young people, they call and inform us about everything that comes around. Like for us [young people], we take them as our parents. They are like a mother, a father, and an aunt.*

However, while many participants were very open to peer supporters, some did not want to disclose personal challenges to them, fearing that this could affect their relationship with the peer supporter. A 23-year-old male living with HIV disclosed his HIV status to the interviewer but had not yet confided in the peer supporter.

*[Interviewer] let me tell you the truth that I even feared to tell my friend [peer supporter] who brought me here. I am HIV positive and on treatment but I face challenges picking treatment from the health facility. I take it [anti-retroviral therapy] on time but I face challenges because it is very far.*

He feared that revealing his status would affect his friendship with the peer supporter and potentially lead to societal stigma, because the peer supporter lived in the same community as him. When the interviewer explained to him that the peer supporters had been trained not only to offer support but also to maintain confidentiality, the young man pledged to meet the peer supporter to get support to access HIV care.

Since the study aimed to test the feasibility and acceptability of the lending a hand intervention, participants were asked about their perceptions of the closure of the intervention, which meant cessation of formal peer support., The responses revealed feelings of loss and disappointment. They said that the intervention had been valuable to young people and they were concerned about the likelihood of many young people who were benefiting from the intervention meeting the same challenges they encountered before they joined. Some young people made heartfelt requests for the study not to close. A 22-year-old female said:

*To tell you the truth, if it is possible, it is better this programme [the lending a hand intervention] is kept on permanently. The problem is that I am poor now, I have no money otherwise I would have bought a present for someone who brought up this idea and asked him/her not to stop.*

Another female, aged 20 years old said:

*Of course, I want to continue with you; I do not want to see this programme [the lending a hand intervention] coming to an end soon. I pray we continue working hand in hand to ensure that you keep on supporting us as young people, and continue meeting, communicating, and checking on us.*

However, a few young people expressed gratitude for the impactful support received and they felt they were now empowered to manage on their own after the intervention’s conclusion and they attributed this to the exposure provided by the peer supporters. They reported increased confidence, attributing this to the knowledge and guidance acquired through their interactions with the peer supporters. They committed to extending help to their peers in terms of information on where to find a health facility and the kind of services provided at these facilities.

## Discussion

Our findings highlight the significant role of peer support in providing a trusted connection through which young migrants could gain the capacity and confidence to engage with existing local health services. Our participants recounted how peer support improved their access to a range of health services. The analysis reveals three distinct themes: facilitating access to existing care, offering responsive and person centred support and fostering a social support system, friendship and mentorship. These findings show how peer support can enhance healthcare access for vulnerable populations such as young migrants, by bringing existing services into closer reach of marginalised groups. Our study findings highlight the challenges migrants face such as limited family and social support networks, stigma associated with seeking sexual healthcare and limited awareness of healthcare services in the host community, challenges that have been reported in other studies [14, 27]. The findings provide evidence of how peer support intervention can address these challenges.

Most of the young migrants in this study came from disadvantaged backgrounds with limited education and formal skills. They were engaged in multiple, low-skilled jobs as a means of livelihood. The peer supporters, who were also young people had themselves experienced similar challenges with migration and had engaged in various casual works to make ends meet. Having successfully gone through these experiences, they were uniquely positioned to provide practical assistance, emotional support, and guidance to the young migrants.

We found that language barriers were a hindrance to accessing health services for young migrants, a finding corroborated in other work on migrants and refugees [28–32]. Peer supporters in this study played a crucial role in overcoming this similar challenge when they worked as interpreters when young migrants visited health facilities when they sought medical care. Our findings show that young migrants were concerned about navigating through the health systems on their own and peer supporters helped them, a concern also reported in the research of Pottie, Batista [33] on migrants to Canada. Peer supporters in our study provided information on health services, including crucial information on HIV prevention products such as PrEP and condoms. This is similar to findings from a study in Zambia that emphasized the role of peer support in safeguarding against HIV and other sexually transmitted infections [34]. The flexible, approachable nature of the peer supporters was invaluable to young people, and this highlights the value of the ‘Lending a Hand’ approach of using peer to peer approach as feasible and acceptable model to support diverse categories of young people, including migrants to address gaps in healthcare access.

Other studies have highlighted the positive influence of peer support in providing emotional and practical support to marginalized populations [35, 36]. In our study, peer supporters emerged as friendly role models who inspired a sense of hope and motivation for the future. We have described elsewhere how important having friends can be in helping young people feel at home in a new place and how peer supporters became part of those friendship networks [37]. However, it is important to consider sustainability and continuity of support beyond the intervention period. Participants expressed concerns about the challenges they may face once the peer supporters are no longer available.

One notable aspect highlighted in the study findings is that some young people expressed discomfort with the preferential treatment they were accorded at the health facilities which resulted in being attended to ahead of other patients they found at the health facility. This underscores the importance of understanding young people’s different preferences when supporting them, the same approach is not necessarily appropriate for all. Also, while young people expressed concerns at the closure of the intervention, a few participants exhibited confidence in their ability to manage future situations independently and attributed this confidence to the peer supporters, and also pledged to assist their peers. This highlights both the direct benefits of peer support and the indirect benefits, with participants becoming the providers of support. Future interventions should consider co-creating interventions with participants so they develop locally relevant and context appropriate approaches they will feel comfortable adopting.

Before the intervention, there were no reported forms of support to young migrants. As reported in our formative research, young migrants often struggled with identifying which people to trust and where to seek help [20]. These struggles highlight the contribution of the intervention in demonstrating how to fill the support gap that was faced by young migrants, and this is also evidenced by most participants’ desire for the peer support intervention to continue.

### Limitations and strength of the study

The first limitation was the loss to follow-up among our participants, resulting in fewer interviews during the second round and as a result, we could therefore have missed an opportunity to understand their individual experiences with peer support. Secondly, we were unable to assess the sustainability of access to services after the conclusion of the study.

The strength of the study relates to the way the intervention was developed. A lot of time was invested in collaborating with the young people from the study area to develop an understanding of their concerns to co-design an intervention which could attend to their needs, employing contextually appropriate approaches.

## Conclusion

Overall, participants felt that the concept of peer support was beneficial and created a positive impact on their lives. No participant reported negative impacts of the intervention. Peer supporters served as friendly role models and fostered hope and trust among the young migrant population. While the study had a positive impact, it highlights the need for sustainability and continuity of interventions for migrants. Policymakers and implementers should consider integrating peer support approaches into migrant interventions and other vulnerable populations as a means of addressing key health and social system gaps to improve their health and social outcomes.

## Supporting information

S1 Checklist Inclusivity form. (DOCX)

S1 Table Thematic table. (XLSX)
